# Basic research and clinical applications of bisphosphonates in bone disease: what have we learned over the last 40 years?

**DOI:** 10.1186/1479-5876-11-303

**Published:** 2013-12-11

**Authors:** Xiao-Long Xu, Wen-Long Gou, Ai-Yuan Wang, Yu Wang, Quan-Yi Guo, Qiang Lu, Shi-Bi Lu, Jiang Peng

**Affiliations:** 1 Institute of Orthopedics, Chinese People’s Liberation Army General Hospital, 28 Fuxing Road, Beijing 100853, People’s Republic of China

**Keywords:** Bisphosphonate, Pharmacokinetics, Osteoclast, Osteoblast, Osteoporosis

## Abstract

It is now 40 years since bisphosphonates (BPs) were first used in the clinic. So, it is timely to provide a brief review of what we have learned about these agents in bone disease. BPs are bone-specific and have been classified into two major groups on the basis of their distinct molecular modes of action: amino-BPs and non-amino-BPs. The amino-BPs are more potent and they inhibit farnesyl pyrophosphate synthase (FPPS), a key enzyme of the mavalonate/cholesterol biosynthetic pathway, while the non-amino-BPs inhibit osteoclast activity, by incorporation into non-hydrolyzable analogs of ATP. Both amino-BPs and non-amino-BPs can protect osteoblasts and osteocytes against apoptosis. The BPs are widely used in the clinic to treat various diseases characterized by excessive bone resorption, including osteoporosis, myeloma, bone metastasis, Legg-Perthes disease, malignant hyperparathyroidism, and other conditions featuring bone fragility. This review provides insights into some of the adverse effects of BPs, such as gastric irritation, osteonecrosis of the jaw, atypical femoral fractures, esophageal cancer, atrial fibrillation, and ocular inflammation. In conclusion, this review covers the biochemical and molecular mechanisms of action of BPs in bone, particularly the discovery that BPs have direct anti-apoptotic effects on osteoblasts and osteocytes, and the current situation of BP use in the clinic.

## Introduction

Bone fragility, leading to fractures and disability, is implicated in the pathogenesis of various bone-desorption diseases induced by glucocorticoid excess, sex-steroid deficiency, and tumors. Today, BPs are the first-line treatment for osteoporosis [1], metastatic bone cancer [2], and Legg-Calve-Perthes disease [3]. BPs are bone-specific and have been used widely in the clinic. However, their exact mechanisms of action remain incompletely understood. Moreover, these medications have attracted much attention mainly because their complications and pathophysiological aspects remain unclear.

In the present review, we summarize the biochemical and molecular mechanisms of action of BPs in bone, particularly the discovery of BPs having direct anti-apoptotic effects on osteoblasts and osteocytes [4,5]. The prospects and caveats for the clinical use of BPs are also discussed.

### Pharmacokinetics

#### Absorption

BPs are administered intravenously or orally. Oral BPs are absorbed into the bloodstream from the gastrointestinal lumen by two routes: 1) transcellularly, transported through epithelial cells into the blood, and 2) intercellularly, whereby BPs gain access to the circulation via the tight junctions between the epithelial cells [6].

Bioavailability is a measure of the rate and extent to which a drug reaches the systemic circulation. The oral bioavailability of BPs is low. The widely used amino-BPs have an absorption of ~0.7%, and non-amino-BPs appear to have a slightly higher absorption, of 2–2.5% [7]. Also, oral absorption is impaired in the presence of food and calcium-, magnesium-, or aluminum-containing drinks and is enhanced with elevated gastric pH [6,8,9]. If the drug is taken with a meal, the absorption may be reduced to zero [10]. Thus, food may have a marked influence on the absorption of BPs. For example, patients are recommended to take their daily dose of oral BPs, such as alendronate, at least 30 min before breakfast [11].

### Distribution of BPs

#### Extra-skeletal

Previous studies of radio-labeled compounds showed that BPs are taken up and adsorbed in to bone primarily, but some also goes to soft tissues, such as the liver, kidney, and spleen [12,13]. The distribution of BPs in extra-skeletal tissue differs, with potential differences in plasma protein binding and kidney concentrations. These differences in distribution may explain, in part, the direct effects of BPs on tumor cells in some studies [14].

#### Distribution in bone

The skeletal distribution and retention of BPs are essential to their effects on bone. Although the uptake and distribution of BPs have been investigated extensively, *in vivo* and *in vitro*, knowledge in humans is incomplete. The amount of BP taken up by bone during the first passage is difficult to quantify. Moreover, the precise route of transfer of BPs from the systemic circulation to bone remains elusive. The distribution in bone is not homogeneous, with the use of ^14^C and ^99^mTc-labeled BPs in animals and humans, respectively. Studies investigating the important steps in the distribution of the BPs in properly performed human studies is necessary. Additionally, some evidence suggests that BPs bind preferentially to bones with high turnover. For example, the uptake of BP in the femur neck and spine is higher than in the femur shaft [15,16].

#### Different binding ability

Competitive bone uptake can occur when two or more BPs are co-administered at high doses [17]. For example, a high concentration of etidronate competes with alendronate binding. Bone uptake may also be influenced by age and gender. The bone turnover rate for modeling and remodeling is age-dependent. Some studies have indicated that the bone turnover rates may differ between young male and female rats, but not older male and female animals [18].

#### Elimination

*In vivo*, only the non N-BPs etidronate and clodronate are metabolized intracellularly to cytotoxic adenosine triphosphate (ATP) analogs; most BPs are *not* metabolized [19]. BPs are excreted unchanged in urine, as shown by ^14^C-labeled studies. Moreover, active tubular secretion of BPs may also be important [20]. After they attach to bone, BPs are liberated again only when the bone in which they are present is resorbed. They can then be taken up again by the skeleton or released into blood. Some amount of BPs can be further embedded in bone during continued bone formation. Thus, the half-life of BPs in bone depends on the rate of bone turnover [6,21].

### Cellular mechanisms of action of BPs

#### Effects on osteoblasts

**Figure 1:**
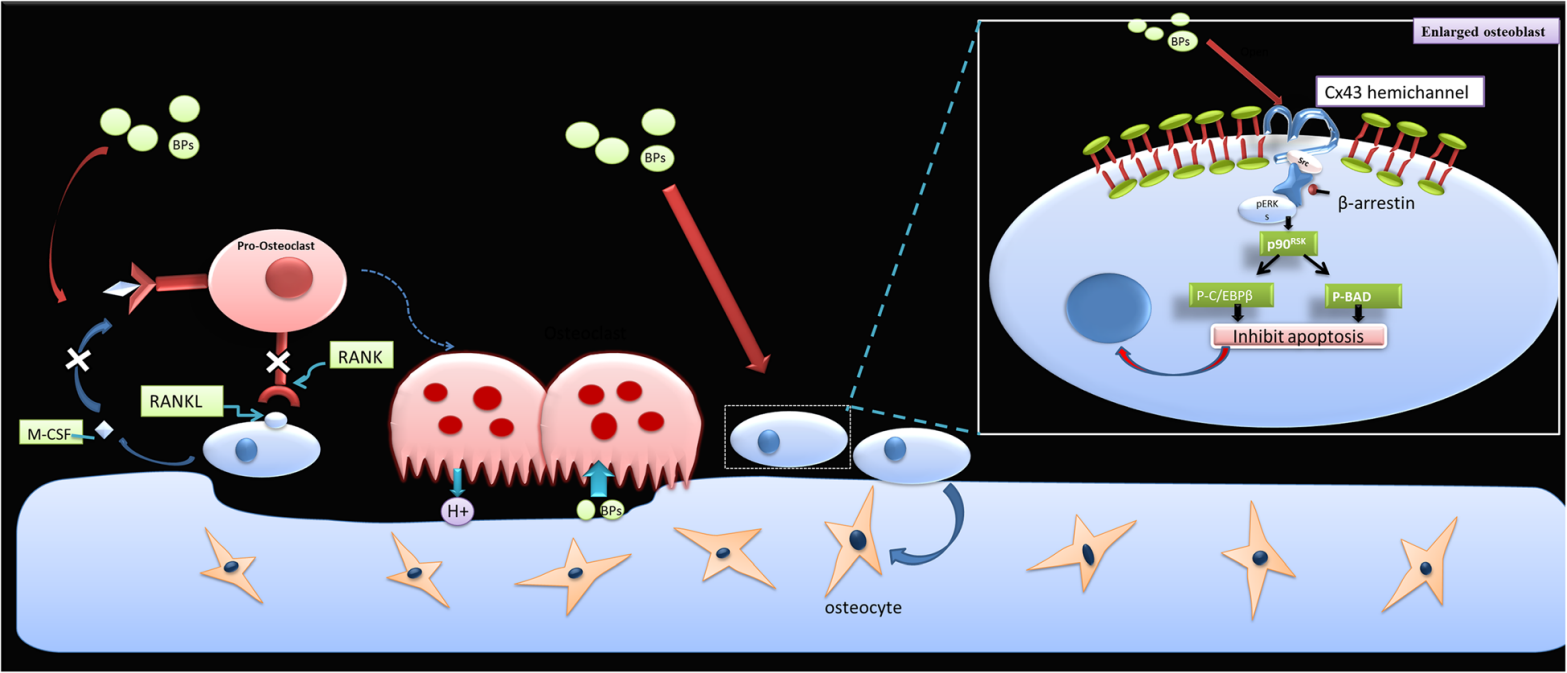
**Effects on osteoblasts.** BPs can down-regulate “receptor activator of NF-κB ligand” (RANKL) and up-regulate osteoprotegerin (OPG) in osteoblasts, indicating indirect effects on the resorption. BPs can inhibit apoptosis of osteoblasts and osteocytes through Cx43 hemichannels. The opening of Cx43 hemichannels results in the activation of kinases, including Src and “extracellular signal-regulated kinases” (ERKs), which initiates the sequential phosphorylation of the ERK cytoplasmic target, p90RSK kinase, and final target substrates, BAD and C/EBPβ, thus suppressing apoptosis.

Despite the well-documented inhibitory effect of BPs on osteoclasts, increasing attention is being focused on their effects on other effector cells, such as osteocytes and osteoblasts. Several early studies showed that BPs could down-regulate “receptor activator of NF-κB ligand” (RANKL) and up-regulate osteoprotegerin (OPG) in osteoblasts, which is one mechanism by which BPs—indirectly—affect resorption [22-26] (Figure 1). Recent studies have shown that BPs affect the expression of OPG and “macrophage colony-stimulating factor” (M-CSF), both essential in osteoclastogenesis (Figure 1). BPs can increase OPG expression and decrease M-CSF expression; in consequence they might inhibit osteoclastogenesis [25]. Substantial evidence has accumulated that BPs modulate the proliferation and differentiation rates of osteoplastic cells, albeit with varying or conflicting effects, in relation to the concentration of BPs [5,27-31]. BPs can promote the growth and differentiation of osteoblasts at lower concentrations, ranging from 10^-9^ to 10^-6^ M but had inhibitory effects at >10^-5^ M [5].

Previous reports have revealed that enhanced viability of osteocytes and osteoblasts may be involved in the beneficial effects of BPs on bone [32]. Early studies showed that BPs suppressed apoptosis in osteocytes and osteoblasts induced by glucocorticoids in mice [33]. Consistently, alendronate has also been shown to exert an inhibitory effect on osteocyte apoptosis induced by fatigue cyclic loading in rats and mice [33-37]. Thus, increasing attention has focused on this and differing mechanisms of action for the anti-apoptotic effects of BPs have been proposed (Figure 1). Recent studies have suggested that the apoptotic effects of BPs depend strictly on the opening of channels formed by connexin43 (Cx43), a member of the connexin family of proteins expressed in osteoblasts and osteocytes [32,33,36-38]. The opening of Cx43 hemichannels results in the activation of kinases, including Src and “extracellular signal-regulated kinases”(ERKs), which initiates the sequential phosphorylation of the ERKs’ cytoplasmic target, p90RSK kinase, and final target substrates, BAD and C/EBPβ, thus suppressing apoptosis [5,33]. Although Cx43 is prerequisite for the prosurvival effect of BPs, recent studies have demonstrated that Cx43 is not required for cell binding of BPs [5,39]. Furthermore, the anti-apoptotic effects of BPs do not depend on inhibitory effects on osteoclasts because analogs that lack anti-resorptive activity could still inhibit apoptosis in osteoblasts and osteocytes without decreasing osteoclast viability [40]. Thus, future studies should address the binding proteins of BPs and new analogs that do not inhibit bone remodeling.

#### Effects on osteoclasts

The significant selectivity of BPs for bone accounts for their efficacy and safety in clinical medicine. Their targeting to bone and their selective uptake by mineral surfaces on bones brings them closely in contact with osteoclasts [4,41]. The uptake of BPs by osteoclasts *in vivo* has been demonstrated using radiolabeling techniques. Previous studies have shown that BPs can affect osteoclast function in various ways, including osteoclast recruitment, differentiation, and resorptive activity, and some may cause apoptosis of osteoclasts [42].

**Figure 2:**
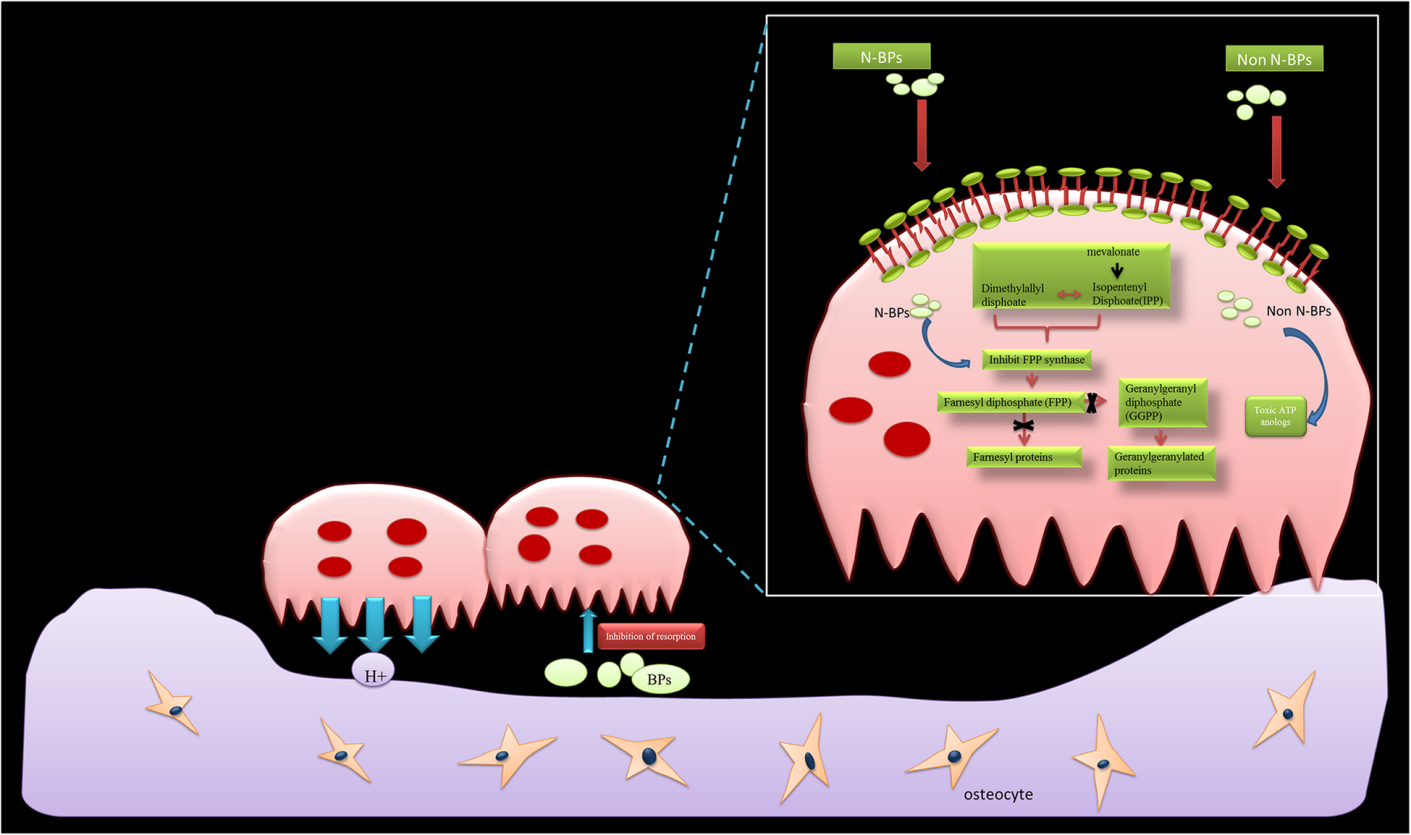
**Effects on osteoclasts.** Osteoclasts release BPs from the bone matrix. N-containing BPs potently inhibit farnesyl pyrophosphate synthase (FPPS), a key enzyme in the mevalonate/cholesterol biosynthetic pathway. Non-N-BPs are incorporated metabolically into non-hydrolyzable cytotoxic analogs of ATP (AppCp).

Currently, BPs are classified into two major groups [4,43,44] on the basis of their distinct molecular mechanisms of action (Figure 2). Members of the first group contain a nitrogen atom; members of this group inhibit the mevalonate biosynthetic pathway, which leads to the synthesis of cholesterol and other sterols. Three major isoprenoid lipids produced in the mevalonate pathway are FPP, isopentenyldiphosphate, and geranyl geranyldiphosphate (GGPP). BPs can inhibit farnesyl pyrophosphate synthase (FPPS), the main enzyme in this pathway [45-47]. FPP and GGPP are required for the prenylation of small GTPases, such as Ras, Rab, Rho, and Rac. Loss of GTPases inhibits the formation of the ruffled border, trafficking of lysosomal enzymes, and transcytosis of degraded bone matrix [48,49].

The second group comprises the non-amino-BPs, such as etidronate and clodronate. Members of this group of BPs can be incorporated metabolically into methylene-containing analogs of ATP [50]. The metabolite analog of ATP is AppCH2p, which contains the P-C-P moiety of medronate in place of the β,γ pyrophosphate (P-O-P) moiety of ATP and results in non-hydrolyzable (AppCp) nucleotides [2,51,52]. Furthermore, the accumulation of AppCp-type metabolites of BPs is associated with cytotoxicity [53-55].

### Clinical applications of bisphosphonates

Forty years have now passed since the first description of BPs [4,56]. BPs have played an important role in the diagnosis and treatment of various diseases during this period. BPs have become crucial for bone imaging and an important treatment for various diseases, such as osteoporosis, myeloma, bone metastasis, Legg-Perthes disease, malignant hyperparathyroidism, and other conditions involving bone fragility [57]. Most of these diseases are characterized by extensive osteoclast activity.

#### Bone scans

According to their diagnostic utility, sensitivity, specificity, and predictive power, BPs were used as agents for bone imaging in the early period. In 1975, ^99m^Tc bone imaging agents were found to be a useful diagnostic method [58,59]. Today, ^99m^Tc diphosphonates are used with ^18^ F fluorodeoxyglucose in metastatic cancer diagnosis because ^99m^Tc has an affinity for sites where bone is actively remolded, while ^18^ F fluorodeoxyglucose is taken up by tumor cells [60,61].

#### Osteoporosis

Today, BPs are an essential first-line therapy for osteoporosis. Early studies showed that BPs could improve bone mineral density (BMD) and decrease the risk of fracture, especially hip fracture [62-68]. Three BPs, alendronate, risedronate, and ibandronate are most widely used in the clinic. Recently, a potent new bisphosphonate, zoledronic acid, has shown high affinity. It can be taken once per year [69,70], which enhances patient willingness to take the medicine. Some studies showed that zoledronic acid had a dose-dependent cytotoxic effect on odontoblast-like cells under clinical conditions [71], drawing attention to the optimal dose and drug “holidays” with these drugs.

#### Anti-cancer

Many kinds of cancers, especially breast, lung and prostate cancers, can metastasize to bone in their disease progression. There are various hypotheses as to how BPs affect tumor cells. Many early studies focused on indirect anti-tumor effects of BPs, the anti-resorptive effects of BPs [72-75]. Recent evidence has shown that BPs can be taken up by other tissues, so they may also have direct effects on tumors [41,76,77]. Some studies showed the BPs could inhibit tumor cell angiogenesis, invasion, proliferation, and survival *in vitro*. For example, zoledronic acid can downregulate the expression of Bcl-2, an anti-apoptotic factor, to induce apoptosis in breast and prostate cancers [76-78]. More recent evidence showed that BPs may inhibit proliferation markers , suppressing the proliferation of tumors [79-84].

#### Bone inflammation diseases

Early studies showed that BPs could be used to suppress the lysis induced by glucocorticoids during rheumatoid arthritis (RA) treatment [85,86]. Recent studies showed that BPs could inhibit some proinflammatory factors, such as interleukin 1(IL-1), IL-6, and tumor necrosis factor-α, [87-91], idicating an anti-inflammatory action of BPs. Also, studies showed that BPs could decrease pain and improve function in osteoarthritis patients [92]. However, the mechanism(s) of these phenomena remain(s) unclear.

### Safety

Despite the widespread use of BPs in the clinic, they have adverse effects, such as gastric irritation, osteonecrosis of the jaw, atypical femoral fractures, esophageal cancer, atrial fibrillation, and ocular inflammation [93].

#### Upper gastrointestinal tract irritation

Gastrointestinal irritation is common with oral BPs, and is the most common reason for treatment discontinuation [94,95]. Alendronate, an oral nitrogen-containing BP, may cause gastrointestinal symptoms such as dyspepsia, esophagitis, esophageal reflux, and gastritis [96].

#### Bisphosphonate-related osteonecrosis of the jaw

Early in the 21st century, the first description of osteonecrosis of the jaw was reported in patients with BP exposure [97]. The adverse effect increased with intravenous injection of BPs [98]. Since then, growing numbers of reports have attempted to describe the effect [65,99-103]. Today, the definition of BRONJ is current or previous treatment with a BP, exposed necrotic bone in the maxillofacial region that has been present for at least 8 weeks, and no history of radiation therapy to the jaws [104,105]. Interestingly, recent studies have shown osteonecrosis of the jaw with the use of denosumab, another anti-resorptive drug [106], and bevacizumab [107-110], an anti-angiogenic agent, so the effect may not be specific to BPs. Thus, many researchers have suggested renaming the condition to “drug-associated osteonecrosis of the jaw.”

Because of the lack of clarity regarding the mechanism, several hypotheses have made to explain how osteonecrosis of the jaw occurs. However, their discoveries share a common feature, infection. Some consider that bone coated with BPs, especially amino-bisphosphonates increases bacterial adhesion, resulting in bone necrosis and osteomyelitis [111-113]. Others consider that inhibition of bone turnover causes necrosis, then infection occurs [114]. Most recently, a study found that osteonecrosis of the jaw can move to adjacent bone and occur in micro-vascular iliac bone grafts used for reconstruction after a partial mandibulectomy [115]. Thus, more research is needed to determine the mechanism of osteonecrosis of the jaw and the connection with BPs.

#### Atypical femoral fracture

Since 2005, increasing numbers of studies of the increased risk of atypical femur fractures in patients taking BPs have been conducted [116-120]. However, later evidence [121,122] showed a lower incidence of atypical femur fractures.

An atypical femoral fracture is located in the femur from just distal to the lesser trochanter to just proximal to the supracondylar flare, associated with no trauma or minimal trauma, a transverse or short oblique configuration, and non-comminuted and complete fractures extending through both cortices with incomplete fractures involving only the lateral cortex [123]. Other features include prodromal pain, increased cortical thickness, bilateral fractures, and delayed healing were also reported [93,124].

How such fractures occur is unknown. Possible mechanisms of BP-related atypical femur fractures include alterations in collagen cross-linking, micro-damage accumulation, increased mineralization, suppression of bone turnover rates, and anti-angiogenic effects [125].

Compared with the considerable benefits of BPs, the incidence of atypical femoral facture is low. Thus, the benefit of continuing therapy may outweigh the possible risk of atypical femoral fracture.

#### Esophageal cancer

In 2009, the US Food and Drug Administration reported the development of esophageal cancer in several patients with a history of oral BP use. Since then, four large databases have been analyzed but conflicting results were reached [114,126,127]. Three of the studies did not find any increased risk, and one found a dose-dependent increased risk of esophageal cancer. Thus, more data are required to assess causality between BPs and esophageal cancer.

#### Atrial fibrillation

An increased incidence of atrial fibrillation was found in the 3-year Health Outcomes and Reduced Incidence with Zoledronic Acid Once Yearly (HORIZON)-Pivotal Fracture Trial of yearly intravenous administration of zoledronate in postmenopausal women with osteoporosis [70]. However, recent studies showed that the risk of atrial fibrillation (AF) or cardiac dysrhythmia was not increased in cancer patients receiving intravenous zoledronic acid [104,128,129]. Moreover, there was no increased risk in postmenopausal women receiving oral alendronate or risedronate [130]. Thus, more effort should be made to discover whether BPs cause an increased risk of atrial fibrillation and, if so, the mechanism(s) of this side effect.

## Conclusions

It is well-established that BPs have become a clinically successful anti-resorptive agent for treating bone disorders. After 40 years of clinical use, the pharmacokinetics of BPs are now clear. They are hardly absorbed through the gastrointestinal lumen , have affinity to the skeleton, and are eliminated slowly. We still have a limited understanding of the cellular mechanism of action of BPs. The biochemical and molecular effects of BPs on osteoclasts can be divided into two distinct mechanisms: direct and indirect. However, the effects on other cell types, such as osteoblast, osteocytes, and monocytes, have not yet been explained fully. Although BPs have been a successful approach to the therapy of bone diseases, exposure to BPs also causes various adverse effects, which have limited their applications. Further studies are required to fully understand the distribution of BPs in extra-skeletal tissues, the effects of BPs on osteocytes, osteoblasts, and monocytes, and to provide new analogs of BPs with fewer limitations in bone turn over and optimal dose and routes of administration. In clinical studies, more attention should be paid to the application of BPs in osteoarthritis patients and in inflammatory bone disease.

## Abbreviations

BPs: Bisphosphonates; FPPS: Farnesyl pyrophosphate synthase; RANKL: Receptor activator of NF-κB ligand; OPG: Osteoprotegerin; M-CSF: Macrophage colony-stimulating factor; ERKs: Extracellular signal-regulated kinases; ATP: Adenosine triphosphate; P-O-P: Pyrophosphate; GGPP: Geranylgeranyl diphosphate; BMD: Bone mineral density; RA: Rheumatoid arthritis; AF: Atrial fibrillation; OA: Osteoarthritis; HORIZON: Health outcomes and reduced incidence with zoledronic acid once yearly.

## Competing interests

The authors declare they have no competing interests.

## Authors’ contributions

XLX, WLG, YW, QYG, AYW and JP reviewed the literature; XLX, SBL, YW and JP wrote the paper; XLX, QL, QYG proofread the final copy. All authors read and approved the final manuscript.
